# Relationship of FDG PET/CT Textural Features with the Tumor Microenvironment and Recurrence Risks in Patients with Advanced Gastric Cancers

**DOI:** 10.3390/cancers14163936

**Published:** 2022-08-15

**Authors:** Hyein Ahn, Geum Jong Song, Si-Hyong Jang, Hyun Ju Lee, Moon-Soo Lee, Ji-Hye Lee, Mee-Hye Oh, Geum Cheol Jeong, Sang Mi Lee, Jeong Won Lee

**Affiliations:** 1 Department of Pathology, Soonchunhyang University Cheonan Hospital, 31 Suncheonhyang 6-gil, Dongnam-gu, Cheonan 31151, Korea; 2 Department of Surgery, Soonchunhyang University Cheonan Hospital, 31 Suncheonhyang 6-gil, Dongnam-gu, Cheonan 31151, Korea; 3 Department of Nuclear Medicine, Soonchunhyang University Cheonan Hospital, 31 Suncheonhyang 6-gil, Dongnam-gu, Cheonan 31151, Korea; 4 Department of Nuclear Medicine, College of Medicine, Catholic Kwandong University, International St. Mary’s Hospital, 25 Simgok-ro 100-gil, Seo-gu, Incheon 22711, Korea

**Keywords:** F-18 fluorodeoxyglucose, positron emission tomography, prognosis, textural feature

## Abstract

**Simple Summary:**

Radiomic analysis using textural features extracted from 2-deoxy-2-[^18^F]fluoro-D-glucose (FDG) positron emission tomography/computed tomography (PET/CT) images was performed to precisely predict metastasis and prognosis in patients with gastric cancer. However, the relationship between FDG PET/CT textural features and histopathological findings in gastric cancer has not been fully evaluated. This study investigated the textural features of gastric cancer on staging FDG PET/CT images with histopathological findings, including components of the immune microenvironment, and recurrence-free survival (RFS) after curative surgery. Textural features were associated with the histopathological classification, Lauren classification, the pN stage of gastric cancer, CD8 T lymphocytes, macrophage infiltrations, and matrix-metalloproteinase-11 expression in the tumor tissue. Textural features were significantly associated with RFS. Textural features of gastric cancer on FDG PET/CT could provide information regarding the histopathological features of cancer cells and the immune microenvironment, and they could be used to predict RFS.

**Abstract:**

The relationship between 2-deoxy-2-[^18^F]fluoro-D-glucose (FDG) positron emission tomography/computed tomography (PET/CT) textural features and histopathological findings in gastric cancer has not been fully evaluated. We investigated the relationship between the textural features of primary tumors on FDG PET/CT with histopathological findings and recurrence-free survival (RFS) in patients with advanced gastric cancer (AGC). Fifty-six patients with AGC who underwent FDG PET/CT for staging work-ups were retrospectively enrolled. Conventional parameters and the first- and second-order textural features of AGC were extracted using PET textural analysis. Upon histopathological analysis, along with histopathological classification and staging, the degree of CD4, CD8, and CD163 cell infiltrations and expressions of interleukin-6 and matrix-metalloproteinase-11 (MMP-11) in the primary tumor were assessed. The histopathological classification, Lauren classification, lymph node metastasis, CD8 T lymphocyte and CD163 macrophage infiltrations, and MMP-11 expression were significantly associated with the textural features of AGC. The multivariate survival analysis showed that increased FDG uptake and intra-tumoral metabolic heterogeneity were significantly associated with an increased risk of recurrence after curative surgery. Textural features of AGC on FDG PET/CT showed significant correlations with the inflammatory response in the tumor microenvironment and histopathological features of AGC, and they showed significant prognostic values for predicting RFS.

## 1. Introduction

The global incidence and mortality rates of gastric cancer have decreased in recent decades; however, gastric cancer remains the third most common cause of cancer-related deaths [1,2]. Based on the depth of tumor invasion, gastric cancer is categorized into early and advanced gastric cancer (AGC), which is defined as cancer that invades beyond the submucosal layer of the stomach wall [3]. In patients with AGC without distant metastasis, adequate resections of primary gastric cancer lesions with regional lymph node dissections are indicated for curative treatment [3]. However, even if patients have received curative surgery, patients with non-metastatic AGC have poor clinical outcomes, with a median overall survival of only 27 months [4]. Therefore, many studies have investigated the prognostic factors of gastric cancer that can be used to predict patient outcomes and select proper treatment strategies [5,6]. In addition to the TNM stage of gastric cancer, the biological characteristics of cancer cells, such as histopathological differentiation and the Lauren classification, have also been suggested as independent predictors for clinical outcomes in patients with AGC [7,8]. Furthermore, several recent studies have found that interactions between gastric cancer cells and their microenvironment play a key role in cancer progression and metastasis [9]. In previous studies investigating the tumor microenvironment, along with immune cells, including T lymphocytes and macrophages, cytokines and proteins involved in the inflammatory response of the microenvironment, such as interleukin-6 (IL-6) and matrix metalloproteinase-11 (MMP-11), showed significant prognostic values in predicting clinical outcomes in patients with gastric cancer [10,11,12]. 

In patients with gastric cancer, 2-deoxy-2-[^18^F]fluoro-D-glucose (FDG) positron emission tomography/computed tomography (PET/CT) showed clinical value in detecting unsuspected metastasis on staging the work-up and recurrence of post-operative surveillance and predicting clinical outcomes [6,13,14]. In previous studies investigating the prognostic significance of FDG PET/CT parameters, maximum FDG uptake and volumetric parameters, such as metabolic tumor volume (MTV) and total lesion glycolysis (TLG), have been generally used as PET imaging parameters of gastric cancer [6,15]. In addition to these conventional PET parameters, several studies have extracted many quantitative textural features from PET images using computational processing methods, such as textural analysis, and evaluated the clinical role of the textural features in patients with gastric cancer [16,17]. The underlying hypothesis regarding the use of textural features is that textural features of medical images can reflect the biological characteristics of cancer tissues, including genomic and proteomic expression patterns, which could provide a large amount of information that is different from traditional clinical factors [5,18]. In previous studies, promising results have been shown in relation to the predictive value of textural features for metastases and clinical outcomes [16,17,19]. However, only a few studies have investigated the relationship between PET/CT textural features and histopathological characteristics of gastric cancer. Furthermore, considering that FDG uptake of cancer lesions is known to be affected by immune cell infiltrations in the tumor tissue [20,21,22], PET/CT textural features might also have a significant relationship with the inflammatory response in the tumor microenvironment, but it has not been fully investigated.

In the present study, we investigated the relationship between the textural features of primary gastric cancer on staging FDG PET/CT images with histopathological findings including components of the tumor immune microenvironment and recurrence-free survival (RFS) after curative surgery in patients with AGC.

## 2. Materials and Methods

### 2.1. Patients

We retrospectively reviewed the medical records of patients who had histopathological diagnoses of gastric cancer and underwent FDG PET/CT for staging work-ups between March 2013 and May 2022 at our medical center. Among them, we enrolled 56 patients who (1) were diagnosed with AGC, (2) showed no distant metastatic lesions on staging imaging studies, and (3) underwent curative surgical resection for the treatment of AGC. We excluded patients who (1) were diagnosed with early gastric cancer, (2) showed distant metastasis on staging examinations or peritoneal seeding metastases on surgical exploration, (3) received any kind of treatment before the surgery or received palliative treatment, (4) had a history of other malignant diseases, (5) had gastric cancer lesions with low FDG uptake inadequate for tumor lesion segmentation by applying the Nestle’s adaptive threshold method, (6) had very small tumor volumes insufficient for extracting textural features, (7) had inadequate surgical specimens for histopathological analyses, and (8) were lost to follow-ups without recurrence or death. 

All enrolled patients underwent diagnostic examinations for staging work-up, including blood tests, gastroduodenoscopy, contrast-enhanced abdominopelvic CT, and FDG PET/CT, and they subsequently underwent total or subtotal gastrectomy with D2 lymphadenectomy. After curative surgery, adjuvant chemotherapy was recommended based on the histopathological stage and the patient’s clinical condition. Regular follow-up was performed at 6–8 month intervals in the first 3 years after treatment and, afterward, at 10–12 months with surveillance examinations, including blood tests, gastroduodenoscopy, and contrast-enhanced abdominopelvic CT. Cancer recurrence after surgery was diagnosed based on the results of histopathological confirmation or follow-up imaging studies. 

The Institutional Review Board of Soonchunhyang University Cheonan Hospital approved this retrospective study (protocol number: SCHCA 2022-07-015) and waived the requirement to obtain written informed consent from the enrolled patients because of the retrospective nature of the study.

### 2.2. FDG PET/CT Image Analysis 

FDG PET/CT images were acquired using a dedicated PET/CT scanner (Biograph mCT 128 scanner, Siemens Healthineers, Knoxville, TN, USA). All patients fasted for at least 6 h, and their blood glucose levels were <200 mg/dL at the time of FDG administration. Approximately 4.07 MBq/kg of FDG uptake was intravenously injected, and after a 60-min uptake period, PET/CT scanning was performed from the skull base to the proximal thigh. Non-contrast-enhanced CT was performed for attenuation correction (100 mA, 120 kVp, slice thickness 5 mm, and slice increment 2.5 mm), and PET was performed for 1.5 min in each bed position using the three-dimensional acquisition mode. PET images were reconstructed using an ordered subset expectation maximization reconstruction algorithm with a time-of-flight function, point-spread function-based Gauss and Allpass filter algorithm, and attenuation correction (2 iterations, 21 subsets, voxel size of 3.0 × 3.0 × 2.5 mm^3^, and a matrix size of 128 × 128).

**Figure 1:**
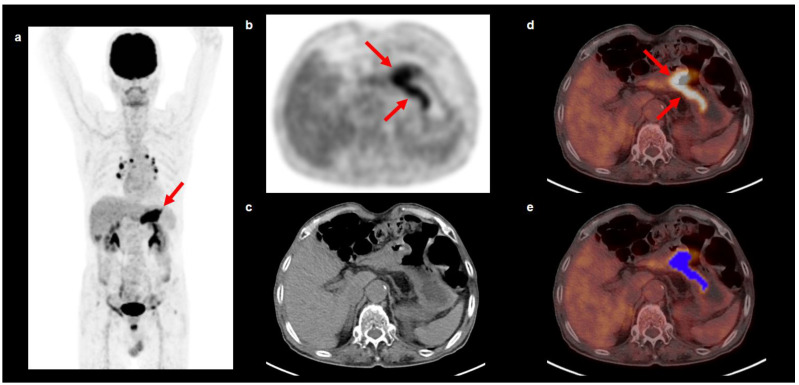
Maximal intensity projection image (**a**) and transaxial PET (**b**), CT (**c**), and fused PET/CT (**d**,**e**) images of FDG PET/CT showing an example of VOI for extracting textural features of gastric cancer. A 76-year-old man underwent FDG PET/CT for staging work-up of gastric cancer in the stomach antrum. The cancer lesion was histopathologically classified as tubular adenocarcinoma, the intestinal type, and showed intensely increased FDG uptake on PET/CT images with a maximum SUV of 8.28 (arrows on (**a**,**b**,**d**)). A VOI was manually drawn around the gastric cancer lesion, and an area that showed a higher SUV than the threshold SUV of 3.45 determined by the modified Nestle’s adaptive threshold method was automatically selected within the VOI (blue color area in (**e**)). The PET/CT textural features of the gastric cancer lesion were extracted from this area.

Tumor segmentation and textural analysis were performed by two experienced nuclear medicine physicians using LIFEx software (version 7.0.0, www.lifexsoft.org, accessed on 5 May 2022) without knowledge of the clinical information and outcomes [23]. A volume-of-interest (VOI) was manually drawn around the AGC lesion on FDG PET/CT images, and the standardized uptake value (SUV) threshold for the delineation of the primary tumor lesion within the VOI was calculated based on the modified Nestle’s adaptive threshold method: (SUV threshold of the primary tumor) = 0.3 × (mean SUV of tumor voxels with FDG uptake >70% of the maximum SUV of the primary tumor) + (mean SUV of the background normal tissue area) [24,25]. The primary gastric cancer lesion that had a greater SUV than the threshold was automatically delineated within the VOI and used in the textural analysis (Figure 1). Delineation of the primary gastric cancer lesion was manually inspected to exclude FDG uptake of the adjacent organs in the textural analysis. From PET images of each primary gastric cancer lesion, a total of 13 textural features, comprising 3 conventional parameters, 4 first-order textural features, and 6 second-order textural features derived from a gray-level co-occurrence matrix (GLCM) were extracted. The 3 conventional parameters consisted of the maximum SUV, MTV, and TLG, and 4 first-order features, including kurtosis, skewness, entropy, and energy were calculated from the SUV histogram. The GLCM was calculated from 13 directions in the 3-dimensional space and 6 GLCM features (contrast, correlation, dissimilarity, energy, entropy, and homogeneity) were measured. 

### 2.3. Histopathological Analysis

**Figure 2:**
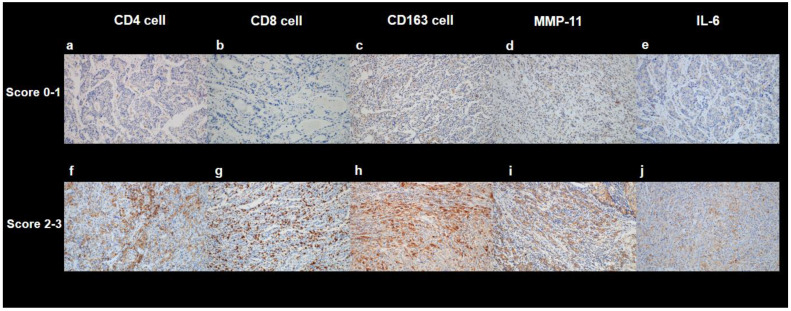
Representative images of immunohistochemical staining of CD4 ((**a**,**f**), ×200), CD8 ((**b**,**g**), ×200), CD163 ((**c**,**h**), ×200), MMP-11 ((**d**,**i**), ×200), and IL-6 ((**e**,**j**), ×200) in the tumor tissue. Examples of a score range of 0-1 are shown in (**a**–**e**), and examples of a score range of 2–3 are shown in (**f**–**j**).

Two experienced pathologists retrospectively analyzed surgical specimens from the patients. The histopathological patterns of gastric cancer were classified into four subtypes: tubular adenocarcinoma, papillary adenocarcinoma, poorly differentiated adenocarcinoma, and signet ring cell carcinoma. None of the enrolled patients had histopathological subtypes other than these 4 subtypes. According to the Lauren classification, the microscopic growth pattern of gastric cancers was categorized into 2 subtypes: intestinal and non-intestinal types. The categories of diffuse, mixed, and non-classifiable types were included in the non-intestinal type [26]. The histopathological stages were determined according to the eighth edition of the American Joint Committee on Cancer Staging guidelines [27]. For immunohistochemical analysis of the tumor tissue, hematoxylin and eosin stained slides made from formalin-fixed, paraffin-embedded tissue blocks were reviewed under a light microscope to prepare fixed tissue specimens that had characteristic cellular morphology and were absent from the necrotic areas. The corresponding areas of each paraffin block were cored twice with a 2 mm-diameter cylinder and assembled into a recipient paraffin block using a tissue microarray (TMA) instrument (Unitma, Seoul, Korea). We performed immunohistochemical staining of individual 4-μm thick slide sections derived from TMA blocks using the Ventana Benchmark XT automated staining system (Ventana Medical Systems, Tucson, AZ, USA) according to the manufacturer’s protocol. The following primary antibodies were used: monoclonal rabbit anti-human CD4 (clone SP35, catalog number 7904423; Ventana Medical System), monoclonal mouse anti-human CD8 (clone C8/144B, catalog number IR623; Dako, Carpinteria, CA, USA), monoclonal mouse anti-human CD163 (clone OTI2G12, catalog number ab156769; Abcam, Cambridge, UK), monoclonal rabbit anti-human MMP-11 (clone SN74-08, catalog number NBP2-67670; Novus Biologicals, Centennial, CO, USA), and polyclonal rabbit anti-human IL-6 (catalog number ab6672; Abcam). All the immunostained slides were blindly evaluated by two experienced pathologists. Three representative areas were selected from each TMA core under a high-power optical microscope (×400 magnification). The numbers of CD4+, CD8+, and CD163+ infiltrating inflammatory cells around the tumor cells were rated as follows: 0, ≤10 cells; 1, 11–50 cells; 2, 51–100 cells; and 3, >100 cells (Figure 2). To score expressions of MMP-11 and IL-6, we used the following intensity score: 0, negative; 1, focal light brown (weak); 2, light brown (moderate); and 3, brown (intense) (Figure 2).

### 2.4. Statistical Analysis

The relationship between the textural features of primary gastric cancer on FDG PET/CT and histopathological findings was evaluated. The Mann–Whitney test was used to compare textural features between patients with intestinal and non-intestinal types and between patients with N0 and N1–N3 stages. The Kruskal–Wallis test was used to evaluate the differences in textural features according to histopathological classification, pT stage, infiltration of CD4, CD8, and CD163 cells, and expressions of MMP-11 and IL-6. For textural parameters that showed statistical significance according to the Kruskal–Wallis test, we further performed a post hoc analysis using Dunn’s test. Univariate and multivariate Cox proportional hazard models were used to investigate the association between textural features of primary gastric cancer and RFS. RFS was defined as the length of time from the date of surgery to the date of detection of gastric cancer recurrence or death. Patients with no events were censored on the date of the last follow-up visit. For continuous variables, optimal cut-off values were selected using the receiver operating characteristic (ROC) curve analysis, and the variables were dichotomized based on the cut-off values. Among the textural parameters of primary gastric cancer, those that showed statistical significance in the univariate analysis were included in the multivariate analysis. In the multivariate analysis, the prognostic values of the selected textural features were evaluated by adding age, sex, and TNM stage as covariates. Textural features that showed significant correlations with each other were assessed using separate models. The Kaplan–Meier method (with the log-rank test) was used to estimate cumulative RFS curves. All statistical analyses were performed using MedCalc Statistical Software version 20.110 (MedCalc Software Ltd., Ostend, Belgium). A *p*-value of <0.05 was regarded as statistically significant.

## 3. Results

### 3.1. Patients’ Clinical Characteristics 

Detailed clinical characteristics of the 56 patients enrolled in this study are summarized in Table 1. In the histopathological analysis, regional lymph node metastasis was found in 38 patients (67.9%), and 32 patients (57.1%) had stage III disease. After curative surgery, 39 patients (69.6%) received adjuvant chemotherapy. The median postoperative follow-up duration was 41.0 months (range, 0.6–102.0 months). During follow-up, 25 patients (44.6%) had events; 21 patients (37.5%) experienced cancer recurrence and 13 patients died (23.2%).

### 3.2. Correlation Analysis between Textural Features and Histopathological Findings

The results of the comparative analysis of FDG PET/CT textural features of primary gastric cancer according to the histopathological findings are shown in Table 2 and Tables S1–S9. Regarding the histopathological classification, patients with papillary/tubular adenocarcinoma had significantly higher values of maximum SUV, SUV histogram entropy, and GLCM correlations than those with signet ring cell carcinoma (*p* < 0.05; Table S1). Concerning the Lauren classification, SUV histogram skewness, GLCM dissimilarity, and GLCM energy showed significant associations, with higher values of GLCM dissimilarity and lower values of SUV histogram skewness and GLCM energy in patients with the intestinal type (*p* < 0.05; Table S2). None of the first-order and second-order textural features showed significant correlations with the T stage (*p* > 0.05; Table S3). Moreover, patients with regional lymph node metastases had significantly higher values of maximum SUV, MTV, TLG, SUV histogram entropy, GLCM contrast, GLCM correlation, and GLCM entropy and lower values of SUV histogram energy, GLCM energy, and GLCM homogeneity than those with no lymph node metastasis (*p* < 0.05; Table S4). 

In the correlation analysis with immunohistochemistry findings of AGC, PET/CT textural features of gastric cancer showed significant associations with the degree of CD8 and CD163 cell infiltrations and MMP-11 expression, whereas none of the textural features showed significant correlations with the degree of CD4 cell infiltration and IL-6 expression (Tables S5–S9). For CD8 cell infiltration, patients with grade 3 showed significantly higher values of maximum SUV, TLG, SUV histogram entropy, GLCM correlation, and GLCM entropy, and significantly lower values of SUV histogram energy and GLCM energy than those with grade 0 (*p* < 0.05; Table S6). For CD163 cell infiltration, patients with grade 3 had significantly higher values of SUV histogram kurtosis, SUV histogram skewness, and GLCM entropy than those with grade 0 (*p* < 0.05; Table S7). Only the GLCM contrast showed a positive relationship with MMP-11 expression in the tumor tissue, revealing significantly higher values in patients with grade 3 than in those with grades 0 and 1 (*p* < 0.05; Table S8).

### 3.3. Survival Analysis of RFS

The association between the PET/CT textural features of primary gastric cancer and RFS was evaluated using Cox regression analysis. All continuous variables included in the analysis were classified into two groups based on the optimal cut-off values selected by the ROC curve analysis. The cut-off values were 65 years for age, 7.53 for maximum SUV, 19.44 cm^3^ for MTV, 56.23 g for TLG, 3.56 for SUV histogram kurtosis, 1.07 for SUV histogram skewness, 0.12 for SUV histogram energy, 3.04 for SUV histogram entropy, 6.41 for GLCM contrast, 0.44 for GLCM correlation, 1.95 for GLCM dissimilarity, 0.02 for GLCM energy, 5.95 for GLCM entropy, and 0.45 for GLCM homogeneity. 

In univariate survival analysis, the maximum SUV, MTV, TLG, SUV histogram energy, SUV histogram entropy, GLCM contrast, GLCM dissimilarity, GLCM energy, GLCM entropy, and GLCM homogeneity were significantly associated with RFS among the PET/CT textural features of primary gastric cancer (*p* < 0.05 for all; Table 3). Additionally, among the clinicopathological factors, the pT stage, pN stage, and TNM stage were significantly associated with RFS (*p* < 0.05 for all).

**Figure 3:**
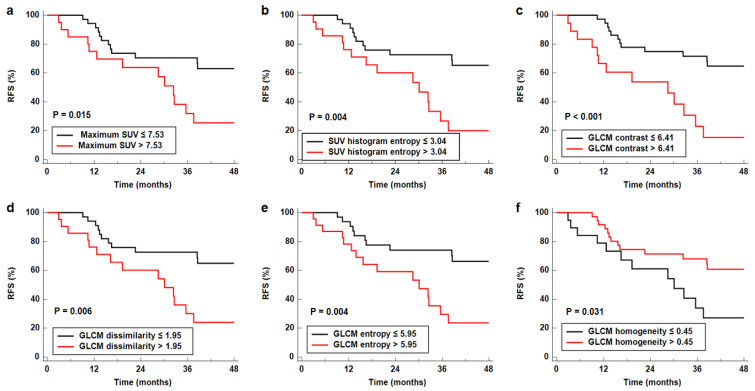
Cumulative RFS curves according to the cut-off values of maximum SUV (**a**), SUV histogram entropy (**b**), GLCM contrast (**c**), GLCM dissimilarity (**d**), GLCM entropy (**e**), and GLCM homogeneity (**f**). *p*-values are the results of the log-rank test.

Textural features that showed statistical significance in the univariate analysis were included in the multivariate survival analysis. Because all textural features included in the multivariate analysis were significantly correlated with one another (*p* < 0.05), the prognostic value of each textural feature was assessed in separate models by adding age, sex, and TNM stage as covariates. The results of the multivariate analysis demonstrated that the maximum SUV, SUV histogram entropy, GLCM contrast, GLCM dissimilarity, GLCM entropy, and GLCM homogeneity remained significant predictors of RFS after adjusting for age, sex, and TNM stage (*p* < 0.05 for all; Table 4). In the Kaplan–Meier analysis, patients with high values of the maximum SUV, SUV histogram entropy, GLCM contrast, GLCM dissimilarity, and GLCM entropy had significantly worse RFS than those with low values (*p* < 0.05; Figure 3a–e), whereas patients with high GLCM homogeneity values showed significantly better RFS than those with low values (*p* = 0.031; Figure 3f).

## 4. Discussion

In this study, we investigated whether the textural features of primary gastric cancer on FDG PET/CT were significantly associated with histopathological findings and RFS in patients with AGC. The correlation analysis with histopathological findings revealed that textural features of gastric cancer were significantly associated not only with histopathological features of gastric cancer cells but also with CD8 T cell and CD163 macrophage infiltrations and MMP-11 expression in the tumor tissue. Moreover, textural features of gastric cancer were found to be significant predictors of RFS after adjusting for age, sex, and TNM stage. 

The significant relationship between the histopathological features of gastric cancer cells and the maximum SUV on FDG PET/CT has already been shown in several studies. Most previous studies demonstrated that the maximum SUVs of gastric cancers with signet ring cell carcinoma histopathology and diffuse types of Lauren classification were significantly lower than those of papillary/tubular adenocarcinoma and intestinal type, respectively [28,29,30]. This finding is considered to result from the distinct histopathological features of signet ring cell carcinoma and diffuse-type gastric cancer, which have a relatively small number of cancer cells with scattered distributions and rich fibrous stroma [30,31]. Furthermore, the distinct molecular and metabolic features of signet ring cell carcinoma, such as low expression levels of glucose transporter-1 and pyruvate kinase, also contribute to low FDG uptake [29]. In the present study, we compared PET/CT textural features, in addition to the maximum SUV, of gastric cancer according to the histopathological features. The first-order features used in this study were calculated from the SUV volume histogram analysis, and SUV histogram kurtosis, skewness, energy, and entropy measure the shape, asymmetry, uniformity, and randomness of the SUV distribution in the SUV histogram, respectively [25]. Second-order features were derived from the GLCM, which reflects the spatial distribution of the SUV intensity levels in a neighborhood [32]. The GLCM energy and GLCM homogeneity measure the uniformity of the SUV distribution in an image, showing high values in images with homogenous or periodic intensity patterns [32]. In contrast, GLCM contrast and dissimilarity measure local variations, and GLCM entropy measures the randomness of the SUV distribution of voxel pairs in an image. High values of these features represent a heterogeneous SUV distribution in the image, indicating increased metabolic heterogeneity [32,33]. On the other hand, the GLCM correlation indicates how the SUV of one voxel correlates well with the SUV of its neighborhood voxels over the whole image, which reflects different aspects of voxel intensity distribution from other GLCM features [32]. In our study, there were significant differences in SUV histogram entropy and GLCM correlation between papillary/tubular adenocarcinoma and signet ring cell carcinoma and significant differences in GLCM dissimilarity and GLCM energy between intestinal and non-intestinal types. Our results indicated a significant difference in intra-tumoral metabolic heterogeneity according to the histopathological features, and probably due to high cellularity and increased metabolic activity, gastric cancer of papillary/tubular adenocarcinoma and intestinal type had more increased intra-tumoral metabolic heterogeneity [29]. Moreover, regional lymph node metastasis of gastric cancer showed significant correlations with PET/CT textural features, demonstrating high intra-tumoral metabolic heterogeneity in gastric cancer lesions with lymph node metastasis. Therefore, in addition to conventional PET/CT parameters, textural features of AGC might have predictive value for histopathological features and lymph node metastasis in patients with AGC [34].

Along with the histopathological features of gastric cancer cells, we also evaluated the relationship between FDG PET/CT textural features and the immune microenvironment in the tumor tissue. Our results demonstrated that the degree of CD8 T lymphocyte and CD 163 macrophage infiltrations and MMP-11 expression were significantly correlated with the textural features of AGC. CD8 T cells and macrophages are the major immune cells in the tumor microenvironment of gastric cancer [35]. Macrophages in the tumor microenvironment predominantly exhibit an M2 phenotype with high CD163 expression [36,37]. M2 macrophages play a crucial role in promoting gastric cancer progression and metastasis and suppressing immune reactions in the tumor microenvironment, revealing a poor prognosis in tumors with increased M2 macrophage infiltration [10,37]. CD8 T lymphocytes are considered to perform important functions in anti-tumor reactions, and several studies have shown an improved prognosis in gastric cancers with a high density of CD8 T lymphocytes [10,38]. In contrast, other studies revealed contradictory results, showing worse survival in patients with high intra-tumoral CD8 T lymphocyte density, and suggested that the effects of CD8 T lymphocytes on cancer cells are dependent on the histopathological features of cancer cells and other immune factors in the tumor microenvironment, such as programmed death-ligand 1 (PD-L1) expression [35,38,39]. MMP-11 is one of the subtypes of MMP, which is a zinc-dependent endopeptidase that degrades the extracellular matrix [11]. Increased expression of MMP-11 is found in both gastric cancer cells and tumor stromal cells, and it plays a significant role in cancer cell invasion and metastasis [11,40]. Considering the significant roles of CD8 T lymphocytes, M2 macrophages, and MMP-11, the results of the present study suggest that textural features of AGC on FDG PET/CT could reflect the degree of immune reaction in the tumor microenvironment, and AGC with increased intra-metabolic heterogeneity could be considered to have a high degree of inflammatory responses in the tumor tissue. However, our study only assessed the relationship between PET/CT features and immunohistochemistry results without investigating the underlying mechanism. Therefore, further studies are needed to determine whether AGC with increased metabolic heterogeneity also had an increased inflammatory response or, conversely, whether increased immune reaction in the tumor tissue contributed to increased intra-tumoral metabolic heterogeneity of AGC. 

Regarding the CD8 T lymphocyte, M2 macrophage, and MMP-11, the CD8 T lymphocyte showed significant associations with the most diverse textural features of AGC. The underlying mechanism of the association between the CD8 T lymphocyte infiltration and PET/CT textural features is unclear. One of the possible explanations is the PD-L1 expression status in the tumor tissue. In a previous study, increased CD8 T lymphocyte infiltration was significantly associated with increased PD-L1 expression in gastric cancer [39]. Furthermore, gastric cancers with positive PD-L1 expressions are found to have higher FDG uptake than those with negative expressions, possibly due to the activations of hypoxia-inducible factor 1-alpha and peroxisome proliferator-activated receptor-gamma, which can upregulate both FDG uptake and PD-L1 expression [41]. Therefore, CD8 T lymphocyte density in gastric cancer could affect maximum SUV and textural features of gastric cancer. Additionally, activated T lymphocytes hyperinduce their glucose uptake and utilization [42]; therefore, activated T lymphocytes could increase FDG uptake and metabolic heterogeneity of the tumor tissue. 

In our study, SUV histogram entropy, GLCM contrast, GLCM dissimilarity, GLCM entropy, and GLCM homogeneity were significant predictors of RFS in the multivariate survival analysis, along with the maximum SUV. In the literature, numerous studies have evaluated the prognostic value of the maximum SUV of gastric cancer for predicting clinical outcomes [26,43], but the prognostic significance of textural features of gastric cancer on FDG PET/CT has been investigated in only a few studies [5,16]. Similar to the results of the present study, a previous study that enrolled 214 patients with stages I–IV gastric cancers showed that the maximum SUV and SUV histogram entropy of gastric cancer lesions were significant predictors for survival, whereas volumetric parameters, including MTV and TLG, did not show statistically significant prognostic values [5]. Considering the significant relationship of PET/CT textural features with the pN stage and inflammatory status of the tumor microenvironment, it might be natural that AGC with high intra-tumoral metabolic heterogeneity had a poor prognosis. In our results, all textural features that were significant predictors for RFS on the multivariate analysis showed correlations with CD8 T lymphocyte infiltration with statistical significance or a marginally significant level, showing increased maximum SUV and intra-tumoral metabolic heterogeneity in patients with increased CD8 T lymphocyte infiltration. Therefore, although, still, contradictory results have been shown regarding the prognostic value of the CD8 T lymphocyte in gastric cancer, our results might be the imaging evidence that increased the CD8 T lymphocyte is related to worse prognosis in patients with AGC. 

The present study has several limitations. First, because this was a retrospective single-center study with a small number of patients, there might be potential bias; therefore, further studies with larger sample sizes are necessary to confirm our results. Second, the prognostic value of the maximum SUV of gastric cancer was shown to differ according to the histopathological classification of gastric cancer, and the histopathological classification of gastric cancer also affects the landscape of the tumor immune microenvironment [29,44]. Hence, it might be necessary to investigate the clinical significance of PET/CT textural features according to the histopathological classification of gastric cancer. Third, the values of PET/CT textural features used in this study are known to be affected by various factors, such as the patient’s body mass index, tumor segmentation method, reconstruction algorithm, and matrix size of PET images [45,46,47]. Finally, to provide a relevant basis for the clinical use of PET/CT textural features, the underlying mechanism of the relationship between textural features and histopathological findings should be further investigated. Especially, considering the significant association of CD8 T lymphocyte infiltration with PET/CT textural features in our results, a more comprehensive analysis of the role of CD8 T lymphocyte in metabolism and progression of gastric cancer should be further investigated. 

## 5. Conclusions

Textural features of AGC on FDG PET/CT showed significant correlations with the histopathological classification, Lauren classification, pN stage of gastric cancer, CD8 T lymphocyte and macrophage infiltrations, and MMP-11 expression in the tumor tissue. Furthermore, these textural features were significantly associated with RFS after adjusting for age, sex, and TNM stage. Increased intra-tumoral metabolic heterogeneity was associated with increased immune reaction in the tumor microenvironment and increased cancer recurrence risk after curative surgical resection. Textural features of AGC on FDG PET/CT could provide information regarding the histopathological features of cancer cells and the immune microenvironment, and they could be used to predict prognosis in patients with AGC. 

## Figures and Tables

**Table 1:** Clinical characteristics of the study population (*n* = 56).

Variables		Number of Patients (%)
Age (years)		Median 59 (range, 34–80)
Sex	Men	35 (62.5%)
	Women	21 (37.5%)
Body mass index (kg/m^2^)		Median 22.6 (range, 16.4–31.5)
Tumor location	Upper	4 (7.1%)
	Middle	23 (41.1%)
	Lower	29 (51.8%)
Histopathological classification	Papillary/tubular adenocarcinoma	34 (60.7%)
	Poorly-differentiated adenocarcinoma	14 (25.0%)
	Signet ring cell carcinoma	8 (14.3%)
Lauren classification	Intestinal	21 (37.5%)
	Non-intestinal	35 (62.5%)
pT stage	T2 stage	14 (25.0%)
	T3 stage	21 (37.5%)
	T4 stage	21 (37.5%)
pN stage	N0 stage	18 (32.1%)
	N1–N3 stage	38 (67.9%)
TNM stage	Stage I	8 (14.3%)
	Stage II	16 (28.6%)
	Stage III	32 (57.1%)
CD4 cell infiltration	Grade 0	14 (25.0%)
	Grade 1	18 (32.1%)
	Grade 2	15 (26.8%)
	Grade 3	9 (16.1%)
CD8 cell infiltration	Grade 0	10 (17.9%)
	Grade 1	11 (19.6%)
	Grade 2	19 (33.9%)
	Grade 3	16 (28.6%)
CD163 cell infiltration	Grade 0	16 (28.6%)
	Grade 1	17 (30.4%)
	Grade 2	12 (21.4%)
	Grade 3	11 (19.6%)
MMP-11 expression	Grade 0	17 (30.4%)
	Grade 1	20 (35.7%)
	Grade 2	12 (21.4%)
	Grade 3	7 (12.5%)
IL-6 expression	Grade 0	19 (33.9%)
	Grade 1	18 (32.1%)
	Grade 2	14 (25.0%)
	Grade 3	5 (8.9%)
Adjuvant chemotherapy	No	17 (30.4%)
	Yes	39 (69.6%)
Follow-up duration (months)		Median 41.0 (range, 0.6–102.0)
Event	Yes (recurrence and/or death)	25 (44.6%)
	No	31 (55.4%)

IL-6, interleukin-6; MMP-11, matrix metalloproteinase-11.

**Table 2:** Statistical significance (*p*-value) of comparisons of textural features of primary gastric cancer on FDG PET/CT according to the histopathological results.

Textural Features	Histopathological Classification *	Lauren Classification ^†^	pT Stage *	pN Stage ^†^	CD4 Cell Infiltration *	CD8 Cell Infiltration *	CD163 Cell Infiltration *	MMP-11 Expression *	IL-6 Expression *
Conventional parameters									
Maximum SUV	0.032	0.168	0.624	0.014	0.088	0.010	0.062	0.221	0.644
MTV	0.908	0.826	0.002	0.017	0.585	0.109	0.933	0.932	0.919
TLG	0.602	0.703	0.024	0.017	0.432	0.021	0.668	0.977	0.788
First-order textural features									
SUV histogram kurtosis	0.322	0.077	0.202	0.079	0.328	0.682	0.008	0.375	0.542
SUV histogram skewness	0.081	0.032	0.665	0.136	0.595	0.408	0.007	0.507	0.525
SUV histogram energy	0.167	0.087	0.407	0.038	0.253	0.026	0.098	0.171	0.381
SUV histogram entropy	0.047	0.122	0.593	0.024	0.313	0.019	0.062	0.193	0.571
Second-order textural features									
GLCM contrast	0.323	0.092	0.843	0.035	0.372	0.077	0.249	0.022	0.229
GLCM correlation	0.036	0.582	0.061	0.016	0.186	0.012	0.090	0.854	0.351
GLCM dissimilarity	0.084	0.015	0.881	0.092	0.691	0.069	0.480	0.069	0.528
GLCM energy	0.061	0.016	0.594	0.018	0.333	0.022	0.136	0.135	0.405
GLCM entropy	0.224	0.122	0.673	0.012	0.268	0.036	0.048	0.120	0.322
GLCM homogeneity	0.076	0.080	0.825	0.044	0.323	0.051	0.062	0.067	0.521

GLCM, gray-level co-occurrence matrix; IL-6, interleukin-6; MMP-11, matrix metalloproteinase-11; MTV, metabolic tumor volume; SUV, standardized uptake value; TLG, total lesion glycolysis. * Results of the Kruskal–Wallis test. ^†^ Results of the Mann–Whitney test.

**Table 3:** Univariate analysis for predicting RFS.

Variables		*p*-Values *	Hazard Ratio (95% Confidence Interval)
Age (≤65 years vs. >65 years)		0.796	1.11 (0.50–2.45)
Sex (women vs. men)		0.275	1.73 (0.65–4.60)
Histopathological classification (papillary/tubular adenocarcinoma vs.)	Poorly-differentiated adenocarcinoma	0.464	1.39 (0.58–3.31)
	Signet ring cell carcinoma	0.933	0.95 (0.27–3.30)
Lauren classification (intestinal vs. non-intestinal)		0.751	0.97 (0.44–2.17)
pT stage (T2 stage vs.)	T3 stage	0.083	6.29 (0.79–50.30)
	T4 stage	0.005	18.69 (2.46–141.80)
pN stage (N0 stage vs. N1–3 stage)		0.005	8.11 (1.91–34.56)
TNM stage (stage I–II vs. stage III)		<0.001	6.68 (2.28–19.63)
Adjuvant treatment (Yes vs. No)		0.071	2.79 (0.92–8.12)
Conventional parameter	Maximum SUV (≤7.53 vs. >7.53)	0.019	2.59 (1.17–5.70)
	MTV (≤19.44 cm^3^ vs. >19.44 cm^3^)	0.002	3.68 (1.65–8.23)
	TLG (≤56.23 g vs. >56.23 g)	0.002	3.77 (1.66–8.55)
First-order textural feature	SUV histogram kurtosis (≤3.56 vs. >3.56)	0.117	1.50 (0.51–3.89)
	SUV histogram skewness (≤1.07 vs. >1.07)	0.117	1.90 (0.85–4.25)
	SUV histogram energy (≤0.12 vs. >0.12)	0.007	0.33 (0.15–0.74)
	SUV histogram entropy (≤3.04 vs. >3.04)	0.006	3.10 (1.39–6.93)
Second-order textural feature	GLCM contrast (≤6.41 vs. >6.41)	<0.001	3.86 (1.73–8.59)
	GLCM correlation (≤0.44 vs. >0.44)	0.178	1.83 (0.76–4.39)
	GLCM dissimilarity (≤1.95 vs. >1.95)	0.009	2.89 (1.30–6.41)
	GLCM energy (≤0.02 vs. >0.02)	0.034	0.42 (0.19–0.94)
	GLCM entropy (≤5.95 vs. >5.95)	0.007	3.08 (1.37–6.94)
	GLCM homogeneity (≤0.45 vs. >0.45)	0.036	0.43 (0.20–0.95)

GLCM, gray-level co-occurrence matrix; MTV, metabolic tumor volume; RFS, recurrence-free survival; SUV, standardized uptake value; TLG, total lesion glycolysis. * Results of the Cox proportional hazards regression analysis.

**Table 4:** Multivariate analysis of textural features for predicting RFS after adjustment for age, sex, and TNM stage.

Variables		*p*-Value *	Hazard Ratio (95% Confidence Interval)
Convention parameter	Maximum SUV (≤7.53 vs. >7.53)	0.029	2.99 (1.12–7.99)
	MTV (≤19.44 cm^3^ vs. >19.44 cm^3^)	0.072	2.20 (0.91–5.50)
	TLG (≤56.23 g vs. >56.23 g)	0.078	2.36 (0.91–6.14)
First-order textural feature	SUV histogram energy (≤0.12 vs. >0.12)	0.086	0.49 (0.22–1.11)
	SUV histogram entropy (≤3.04 vs. >3.04)	0.033	3.04 (1.09–8.45)
Second-order textural feature	GLCM contrast (≤6.41 vs. >6.41)	0.006	3.88 (1.47–10.22)
	GLCM dissimilarity (≤1.95 vs. >1.95)	0.007	4.14 (1.49–11.53)
	GLCM energy (≤0.02 vs. >0.02)	0.167	0.50 (0.19–1.33)
	GLCM entropy (≤5.95 vs. >5.95)	0.020	3.19 (1.20–8.47)
	GLCM homogeneity (≤0.45 vs. >0.45)	0.046	0.37 (0.14–0.98)

GLCM, gray-level co-occurrence matrix; MTV, metabolic tumor volume; RFS, recurrence-free survival; SUV, standardized uptake value; TLG, total lesion glycolysis. * Results of the Cox proportional hazards regression analysis.

## Data Availability

The datasets generated during and/or analyzed during the current study are available from the corresponding authors upon reasonable request.

## Supplementary Materials

The following supporting information can be downloaded at: https://www.mdpi.com/article/10.3390/cancers14163936/s1, Table S1: Comparisons of textural features of primary gastric cancer on FDG PET/CT according to the histopathological classification; Table S2: Comparisons of textural features of primary gastric cancer on FDG PET/CT according to the Lauren classification; Table S3: Comparisons of textural features of primary gastric cancer on FDG PET/CT according to pT stage; Table S4: Comparisons of textural features of primary gastric cancer on FDG PET/CT according to pN stage; Table S5: Comparisons of textural features of primary gastric cancer on FDG PET/CT according to CD4 cell infiltration in the tumor; Table S6: Comparisons of textural features of primary gastric cancer on FDG PET/CT according to CD8 cell infiltration in the tumor; Table S7: Comparisons of textural features of primary gastric cancer on FDG PET/CT according to CD163 cell infiltration in the tumor; Table S8: Comparisons of textural features of primary gastric cancer on FDG PET/CT according to matrix metalloproteinase-11 (MMP-11) expression in the tumor; Table S9: Comparisons of textural features of primary gastric cancer on FDG PET/CT according to interleukin-6 (IL-6) expression in the tumor.
