# Supplementary material for: Relationship of FDG PET/CT Textural Features with the Tumor Microenvironment and Recurrence Risks in Patients with Advanced Gastric Cancers
Source: Cancers (Basel). 2022 Aug 15;14(16):3936. doi: 10.3390/cancers14163936 (PMC9406203; doi:10.3390/cancers14163936)
Supplement: Supplementary file 1 [file cancers-14-03936-s001.zip › cancers-1840097-supplementary.pdf]

Article title: Relationship of FDG PET/CT textural features with tumor microenvironment and recurrence risk in patients with advanced gastric cancers

Authors: Hyein Ahn, Geum Jong Song, Si-Hyong Jang, Hyun Ju Lee, Moon-Soo Lee, Ji-Hye Lee, Mee-Hye Oh, Geum Cheol Jeong, Sang Mi Lee, Jeong Won Lee

Table S1. Comparisons of textural features of primary gastric cancer on FDG PET/CT according to the histopathological classification.

Textural features	Papillary/tubular adenocarcinoma	Poorly-differentiated adenocarcinoma	Signet ring cell carcinoma	P-value*
Conventional parameters				
Maximum SUV	5.91 (4.14–10.53)	4.93 (3.86–10.66)	3.68 (3.30–5.27)	**0.032†**
MTV	9.71 (6.06–19.61)	8.44 (6.22–21.48)	9.61 (4.27–19.22)	0.908
TLG	42.28 (16.65–109.98)	37.94 (18.22–86.46)	26.73 (10.96–55.20)	0.602
First-order textural features				
SUV histogram kurtosis	3.12 (2.60–3.68)	3.16 (2.14–3.87)	2.62 (2.43–3.05)	0.322
SUV histogram skewness	0.85 (0.64–1.08)	0.95 (0.73–1.23)	0.63 (0.42–0.72)	0.081
SUV histogram energy	0.17 (0.08–0.26)	0.19 (0.09–0.37)	0.30 (0.23–0.34)	0.167
SUV histogram entropy	2.79 (2.21–3.82)	2.59 (1.95–3.86)	1.85 (1.61–2.16)	**0.047†**
Second-order textural features				
GLCM contrast	3.10 (1.70–12.65)	3.38 (0.91–17.78)	1.43 (1.08–7.54)	0.323
GLCM correlation	0.54 (0.41–0.62)	0.49 (0.17–0.60)	0.31 (0.27–0.37)	**0.036†**
GLCM dissimilarity	1.64 (1.00–3.02)	1.36 (0.67–3.18)	0.81 (0.75–0.99)	0.084
GLCM energy	0.03 (0.01–0.06)	0.04 (0.02–0.10)	0.11 (0.07–.12)	0.061
GLCM entropy	5.33 (4.40–7.28)	5.61 (3.46–7.00)	3.80 (3.23–5.59)	0.224
GLCM homogeneity	0.51 (0.33–0.62)	0.55 (0.35–0.71)	0.65 (0.62–0.67)	0.076

GLCM = grey-level co-occurrence matrix; MTV = metabolic tumor volume; SUV = standardized uptake value; TLG = total lesion glycolysis

Expressed in median value (25 percentile – 75 percentile)

*Results of the Kruskal–Wallis test

†On post-hoc analysis, patients with papillary/tubular adenocarcinoma had significantly higher values of parameters than those with signet ring cell carcinoma ($p<0.05$)

Table S2. Comparisons of textural features of primary gastric cancer on FDG PET/CT according to the Lauren classification.

Textural features	Intestinal type	Non-intestinal type	P-value*
Conventional parameters			
Maximum SUV	6.54 (3.88–10.71)	4.90 (3.38–8.63)	0.168
MTV	7.66 (5.57–22.83)	10.71 (6.18–19.25)	0.826
TLG	41.39 (16.62–124.10)	39.54 (17.82–82.58)	0.703
First-order textural features			
SUV histogram kurtosis	2.85 (2.43–3.24)	3.15 (2.60–3.82)	0.077
SUV histogram skewness	0.70 (0.58–0.91)	0.85 (0.66–1.10)	**0.032**
SUV histogram energy	0.14 (0.08–0.25)	0.22 (0.13–0.34)	0.087
SUV histogram entropy	3.04 (2.25–3.92)	2.32 (1.83–3.43)	0.122
Second-order textural features			
GLCM contrast	5.38 (1.96–17.11)	2.73 (1.05–10.17)	0.092
GLCM correlation	0.48 (0.36–0.55)	0.53 (0.28–0.62)	0.582
GLCM dissimilarity	2.64 (1.09–4.89)	1.24 (0.75–2.18)	**0.015**
GLCM energy	0.03 (0.01–0.06)	0.06 (0.02–0.11)	**0.016**
GLCM entropy	5.95 (4.47–7.51)	4.74 (3.47–6.56)	0.122
GLCM homogeneity	0.47 (0.34–0.60)	0.57 (0.40–0.67)	0.080

GLCM = grey-level co-occurrence matrix; MTV = metabolic tumor volume; SUV = standardized uptake value; TLG = total lesion glycolysis

Expressed in median value (25 percentile – 75 percentile)

*Results of the Mann–Whitney test

Table S3. Comparisons of textural features of primary gastric cancer on FDG PET/CT according to pT stage.

Textural features	T2 stage	T3 stage	T4 stage	P-value*
Conventional parameters				
Maximum SUV	4.24 (3.43–6.54)	5.02 (3.59–10.61)	5.42 (4.07–8.74)	0.624
MTV	6.04 (4.23–7.66)	8.70 (5.78–18.44)	19.61 (8.17–30.30)	**0.002†**
TLG	17.11 (10.75–43.98)	40.01 (19.14–58.76)	76.63 (29.35–118.22)	**0.024†**
First-order textural features				
SUV histogram kurtosis	2.86 (2.47–3.18)	2.87 (2.46–3.76)	3.45 (2.65–3.93)	0.202
SUV histogram skewness	0.82 (0.66–0.94)	0.79 (0.58–1.09)	0.85 (0.68–1.10)	0.665
SUV histogram energy	0.27 (0.14–0.35)	0.17 (0.08–0.26)	0.18 (0.09–0.31)	0.407
SUV histogram entropy	2.23 (1.67–3.04)	2.78 (1.98–3.58)	2.80 (1.97–3.74)	0.593
Second-order textural features				
GLCM contrast	2.70 (1.14–6.15)	3.29 (1.55–12.80)	3.46 (1.67–14.90)	0.843
GLCM correlation	0.37 (0.15–0.54)	0.52 (0.39–0.61)	0.53 (0.35–0.64)	0.061
GLCM dissimilarity	1.24 (0.81–1.95)	1.38 (0.93–2.79)	1.40 (0.91–3.10)	0.881
GLCM energy	0.06 (0.02–0.12)	0.03 (0.01–0.08)	0.04 (0.02–0.09)	0.594
GLCM entropy	4.64 (3.37–6.62)	5.38 (3.94–7.20)	5.27 (3.93–7.12)	0.673
GLCM homogeneity	0.57 (0.46–0.65)	0.53 (0.32–0.63)	0.54 (0.38–0.64)	0.825

GLCM = grey-level co-occurrence matrix; MTV = metabolic tumor volume; SUV = standardized uptake value; TLG = total lesion glycolysis

Expressed in median value (25 percentile – 75 percentile)

*Results of the Kruskal–Wallis test

†On post-hoc analysis, patients with T4 stage had significantly higher values of parameters than those with T2 stage ($p<0.05$)

Table S4. Comparisons of textural features of primary gastric cancer on FDG PET/CT according to pN stage.

Textural features	N0 stage	N1–N3 stage	P-value*
Conventional parameters			
Maximum SUV	3.69 (3.30–6.30)	5.91 (4.13–10.53)	**0.014**
MTV	6.14 (4.52–15.47)	12.93 (6.37–22.09)	**0.017**
TLG	20.67 (10.98–39.54)	44.15 (23.24–109.98)	**0.017**
First-order textural features			
SUV histogram kurtosis	2.76 (2.36–3.23)	3.12 (2.57–3.81)	0.079
SUV histogram skewness	0.80 (0.56–0.94)	0.85 (0.64–1.10)	0.136
SUV histogram energy	0.26 (0.18–0.37)	0.16 (0.09–0.26)	**0.038**
SUV histogram entropy	1.97 (1.57–2.81)	2.88 (2.21–3.82)	**0.024**
Second-order textural features			
GLCM contrast	1.42 (0.91–6.15)	4.10 (1.96–14.43)	**0.035**
GLCM correlation	0.39 (0.15–0.53)	0.54 (0.36–0.62)	**0.016**
GLCM dissimilarity	0.91 (0.67–1.95)	1.39 (0.99–3.07)	0.092
GLCM energy	0.09 (0.03–0.13)	0.03 (0.01–0.06)	**0.018**
GLCM entropy	3.75 (3.10–5.45)	5.99 (4.55–7.28)	**0.012**
GLCM homogeneity	0.63 (0.47–0.70)	0.51 (0.35–0.62)	**0.044**

GLCM = grey-level co-occurrence matrix; MTV = metabolic tumor volume; SUV = standardized uptake value; TLG = total lesion glycolysis

Expressed in median value (25 percentile – 75 percentile)

*Results of the Mann–Whitney test

Table S5. Comparisons of textural features of primary gastric cancer on FDG PET/CT according to CD4 cell infiltration in the tumor.

Textural features	Grade 0	Grade 1	Grade 2	Grade 3	P-value*
Conventional parameters					
Maximum SUV	4.27 (3.30–5.42)	6.05 (3.35–11.66)	5.09 (3.89–7.31)	8.81 (5.58–12.38)	0.088
MTV	8.19 (4.99–15.47)	9.53 (6.14–22.09)	12.99 (6.35–28.04)	7.11 (5.45–18.49)	0.585
TLG	27.60 (11.54–41.39)	41.99 (15.39–142.48)	43.98 (24.49–98.35)	44.33 (15.18–134.76)	0.432
First-order textural features					
SUV histogram kurtosis	2.77 (2.47–3.49)	2.73 (2.36–3.61)	3.37 (2.95–3.65)	3.04 (2.81–3.98)	0.328
SUV histogram skewness	0.71 (0.56–1.07)	0.74 (0.62–1.09)	0.85 (0.67–1.07)	0.86 (0.76–1.24)	0.595
SUV histogram energy	0.26 (0.18–0.37)	0.18 (0.08–0.35)	0.17 (0.12–0.25)	0.16 (0.06–0.25)	0.253
SUV histogram entropy	2.16 (1.84–2.81)	2.76 (1.54–4.27)	2.78 (2.21–3.38)	2.96 (2.29–4.32)	0.313
Second-order textural features					
GLCM contrast	1.84 (1.02–4.75)	6.21 (0.73–16.30)	3.06 (1.63–5.96)	6.41 (1.88–35.16)	0.372
GLCM correlation	0.37 (0.27–0.53)	0.43 (0.21–0.62)	0.53 (0.46–0.61)	0.55 (0.44–0.59)	0.186
GLCM dissimilarity	1.02 (0.74–2.64)	1.66 (0.59–3.07)	1.30 (0.96–2.65)	1.91 (1.00–4.70)	0.691
GLCM energy	0.07 (0.03–0.11)	0.04 (0.01–0.16)	0.04 (0.02–0.07)	0.03 (0.01–0.06)	0.333
GLCM entropy	4.32 (3.46–5.45)	5.46 (3.09–7.40)	5.38 (4.67–6.56)	5.85 (4.50–7.93)	0.268
GLCM homogeneity	0.62 (0.48–0.67)	0.49 (0.38–0.72)	0.54 (0.33–0.62)	0.45 (0.28–0.61)	0.323

GLCM = grey-level co-occurrence matrix; MTV = metabolic tumor volume; SUV = standardized uptake value; TLG = total lesion glycolysis

Expressed in median value (25 percentile – 75 percentile)

*Results of the Kruskal–Wallis test

Table S6. Comparisons of textural features of primary gastric cancer on FDG PET/CT according to CD8 cell infiltration in the tumor.

Textural features	Grade 0	Grade 1	Grade 2	Grade 3	P-value*
Conventional parameters					
Maximum SUV	3.73 (3.35–4.90)	7.03 (3.72–10.80)	4.51 (3.41–5.68)	9.47 (5.14–12.62)	**0.010**†
MTV	7.43 (4.98–16.27)	16.95 (8.83–21.46)	6.22 (4.04–12.42)	17.30 (6.46–21.69)	0.109
TLG	27.61 (11.54–39.54)	48.23 (21.02–136.68)	21.50 (10.57–42.58)	57.14 (34.89–136.05)	**0.021**†
First-order textural features					
SUV histogram kurtosis	3.28 (2.44–3.92)	2.67 (2.22–3.35)	2.96 (2.58–4.23)	3.15 (2.58–3.71)	0.682
SUV histogram skewness	0.95 (0.69–1.10)	0.70 (0.47–0.91)	0.74 (0.63–0.96)	0.92 (0.63–1.15)	0.408
SUV histogram energy	0.32 (0.22–0.40)	0.18 (0.08–0.30)	0.23 (0.14–0.33)	0.14 (0.06–0.21)	**0.026**‡
SUV histogram entropy	1.92 (1.54–2.49)	2.89 (2.04–3.85)	2.26 (1.72–3.11)	3.23 (2.40–4.89)	**0.019**†
Second-order textural features					
GLCM contrast	1.41 (0.91–2.74)	5.38 (1.59–15.39)	3.03 (1.25–6.15)	8.04 (2.31–43.90)	0.077
GLCM correlation	0.38 (0.15–0.53)	0.55 (0.47–0.61)	0.36 (0.26–0.60)	0.57 (0.48–0.66)	**0.012**§
GLCM dissimilarity	0.88 (0.67–1.26)	2.64 (0.88–4.93)	1.30 (0.78–1.93)	2.08 (1.12–5.10)	0.069
GLCM energy	0.11 (0.05–0.14)	0.04 (0.01–0.09)	0.06 (0.03–0.10)	0.02 (0.01–0.05)	**0.022**‡
GLCM entropy	3.79 (3.09–4.93)	5.13 (3.84–6.71)	4.59 (3.52–6.35)	6.32 (4.75–8.08)	**0.036**†
GLCM homogeneity	0.64 (0.55–0.71)	0.52 (0.38–0.65)	0.59 (0.46–0.67)	0.44 (0.28–0.58)	0.051

GLCM = grey-level co-occurrence matrix; MTV = metabolic tumor volume; SUV = standardized uptake value; TLG = total lesion glycolysis

Expressed in median value (25 percentile – 75 percentile)

*Results of the Kruskal–Wallis test

†On post-hoc analysis, patients with grade 3 had significantly higher values of parameters than those with grade 0 ($p<0.05$)

‡On post-hoc analysis, patients with grade 0 had significantly higher values of parameters than those with grade 3 ($p<0.05$)

§On post-hoc analysis, patients with grade 3 had significantly higher values of parameter than those with grade 0 and 2 ($p<0.05$)

Table S7. Comparisons of textural features of primary gastric cancer on FDG PET/CT according to CD163 cell infiltration in the tumor.

Textural features	Grade 0	Grade 1	Grade 2	Grade 3	P-value*
Conventional parameters					
Maximum SUV	4.39 (3.35–7.55)	5.02 (3.65–8.13)	5.69 (3.82–8.44)	8.81 (6.50–11.71)	0.062
MTV	10.04 (5.60–18.82)	7.67 (5.74–22.52)	8.00 (5.15–20.67)	10.71 (6.21–21.47)	0.933
TLG	35.71 (14.88–84.04)	39.54 (15.23–83.05)	34.48 (16.25–76.41)	47.91 (23.89–127.61)	0.668
First-order textural features					
SUV histogram kurtosis	2.56 (2.34–3.25)	3.18 (2.67–3.96)	2.52 (2.34–3.09)	3.61 (3.07–3.90)	**0.008**†
SUV histogram skewness	0.71 (0.42–1.06)	0.75 (0.66–1.10)	0.85 (0.41–1.14)	1.09 (0.87–1.17)	**0.007**‡
SUV histogram energy	0.27 (0.13–0.37)	0.23 (0.13–0.30)	0.21 (0.12–0.28)	0.12 (0.08–0.15)	0.098
SUV histogram entropy	2.25 (1.71–3.31)	2.38 (1.97–3.28)	2.48 (1.87–3.46)	3.50 (3.02–4.15)	0.062
Second-order textural features					
GLCM contrast	2.36 (0.97–5.06)	2.04 (1.60–15.27)	3.06 (1.40–24.10)	9.67 (3.82–13.09)	0.249
GLCM correlation	0.45 (0.17–0.58)	0.48 (0.27–0.64)	0.41 (0.29–0.55)	0.56 (0.50–0.62)	0.090
GLCM dissimilarity	1.14 (0.71–2.85)	1.25 (0.94–3.06)	1.17 (0.79–3.28)	2.26 (1.45–2.85)	0.480
GLCM energy	0.06 (0.03–0.12)	0.06 (0.02–0.08)	0.05 (0.02–0.11)	0.02 (0.01–0.03)	0.136
GLCM entropy	4.28 (3.28–6.14)	4.29 (3.94–6.16)	5.41 (3.93–6.76)	6.79 (5.41–8.38)	**0.048**§
GLCM homogeneity	0.59 (0.44–0.69)	0.59 (0.38–0.63)	0.48 (0.38–0.56)	0.39 (0.28–0.45)	0.062

GLCM = grey-level co-occurrence matrix; MTV = metabolic tumor volume; SUV = standardized uptake value; TLG = total lesion glycolysis

Expressed in median value (25 percentile – 75 percentile)

*Results of the Kruskal–Wallis test

†On post-hoc analysis, patients with grade 3 had significantly higher values of parameter than those with grade 0 and grade 2 ($p<0.05$)

‡On post-hoc analysis, patients with grade 3 had significantly higher values of parameter than those with grade 0, grade 1, and grade 2 ($p<0.05$)

§On post-hoc analysis, patients with grade 3 had significantly higher values of parameter than those with grade 0 and grade 1 ($p<0.05$)

Table S8. Comparisons of textural features of primary gastric cancer on FDG PET/CT according to matrix metalloproteinase-11 (MMP-11) expression in the tumor.

Textural features	Grade 0	Grade 1	Grade 2	Grade 3	P-value*
Conventional parameters					
Maximum SUV	4.13 (3.30–5.77)	5.59 (3.77–8.55)	5.60 (4.24–9.10)	10.66 (5.08–11.41)	0.221
MTV	10.87 (5.91–21.85)	9.99 (6.11–22.00)	7.67 (6.04–18.89)	8.34 (5.06–18.90)	0.932
TLG	39.45 (16.55–78.79)	42.00 (16.02–121.73)	39.11 (17.11–81.55)	40.82 (21.94–113.73)	0.977
First-order textural features					
SUV histogram kurtosis	2.67 (2.43–3.71)	3.05 (2.51–3.39)	3.50 (2.94–3.72)	2.82 (2.48–3.65)	0.375
SUV histogram skewness	0.72 (0.42–1.08)	0.81 (0.65–0.99)	0.93 (0.82–1.10)	0.74 (0.61–1.15)	0.507
SUV histogram energy	0.28 (0.19–0.37)	0.17 (0.12–0.24)	0.20 (0.11–0.27)	0.08 (0.06–0.27)	0.171
SUV histogram entropy	2.02 (1.77–2.72)	2.79 (2.19–3.55)	2.61 (2.19–3.66)	3.86 (2.29–4.23)	0.193
Second-order textural features					
GLCM contrast	1.70 (0.89–3.65)	3.10 (1.51–8.04)	3.22 (2.12–14.06)	16.30 (13.14–54.95)	**0.022†**
GLCM correlation	0.50 (0.24–0.60)	0.54 (0.36–0.60)	0.43 (0.32–0.54)	0.45 (0.29–0.65)	0.854
GLCM dissimilarity	0.96 (0.66–1.64)	1.38 (0.91–2.51)	1.67 (1.18–3.10)	3.07 (1.47–6.39)	0.069
GLCM energy	0.07 (0.04–0.13)	0.03 (0.02–0.07)	0.04 (0.01–0.06)	0.01 (0.01–0.08)	0.135
GLCM entropy	4.07 (3.37–5.33)	5.37 (4.27–6.46)	6.17 (4.54–6.76)	7.40 (4.67–8.38)	0.120
GLCM homogeneity	0.62 (0.51–0.71)	0.51 (0.38–0.63)	0.55 (0.40–0.60)	0.33 (0.27–0.56)	0.067

GLCM = grey-level co-occurrence matrix; MTV = metabolic tumor volume; SUV = standardized uptake value; TLG = total lesion glycolysis

Expressed in median value (25 percentile – 75 percentile)

*Results of the Kruskal–Wallis test

†On post-hoc analysis, patients with grade 3 had significantly higher values of parameter than those with grade 0 and grade 1 ($p<0.05$)

Table S9. Comparisons of textural features of primary gastric cancer on FDG PET/CT according to interleukin-6 (IL-6) expression in the tumor.

Textural features	Grade 0	Grade 1	Grade 2	Grade 3	P-value*
Conventional parameters					
Maximum SUV	4.51 (3.63–7.00)	5.43 (3.68–8.08)	6.42 (3.45–10.66)	7.11 (4.46–11.00)	0.644
MTV	7.66 (6.15–21.29)	10.27 (4.70–21.90)	14.23 (4.23–19.61)	10.71 (7.81–22.22)	0.919
TLG	24.97 (17.82–63.56)	44.16 (16.65–76.63)	39.73 (11.13–133.48)	46.09 (37.75–94.66)	0.788
First-order textural features					
SUV histogram kurtosis	3.02 (2.48–3.56)	2.91 (2.33–3.74)	3.22 (2.85–3.87)	2.56 (2.46–3.54)	0.542
SUV histogram skewness	0.74 (0.66–1.07)	0.74 (0.58–1.08)	0.95 (0.82–1.24)	0.66 (0.63–0.90)	0.525
SUV histogram energy	0.25 (0.14–0.36)	0.20 (0.12–0.25)	0.16 (0.09–0.32)	0.13 (0.07–0.21)	0.381
SUV histogram entropy	2.26 (1.86–3.26)	2.56 (2.17–3.44)	2.91 (1.87–3.86)	3.22 (2.51–4.10)	0.571
Second-order textural features					
GLCM contrast	2.19 (0.97–5.82)	2.86 (1.60–13.24)	4.65 (1.19–12.65)	13.11 (5.44–29.08)	0.229
GLCM correlation	0.40 (0.23–0.58)	0.51 (0.28–0.57)	0.55 (0.39–0.67)	0.53 (0.23–0.59)	0.351
GLCM dissimilarity	1.11 (0.70–2.46)	1.30 (0.96–3.18)	1.67 (0.81–2.76)	1.94 (1.24–5.71)	0.528
GLCM energy	0.06 (0.03–0.13)	0.05 (0.02–0.07)	0.03 (0.01–0.10)	0.02 (0.01–0.05)	0.405
GLCM entropy	4.49 (3.47–6.27)	5.15 (4.14–6.14)	6.28 (3.89–7.40)	6.40 (5.48–7.13)	0.322
GLCM homogeneity	0.59 (0.41–0.69)	0.57 (0.28–0.62)	0.39 (0.49–0.65)	0.45 (0.31–0.57)	0.521

GLCM = grey-level co-occurrence matrix; MTV = metabolic tumor volume; SUV = standardized uptake value; TLG = total lesion glycolysis

Expressed in median value (25 percentile – 75 percentile)

*Results of the Kruskal–Wallis test
